# Gait Pattern at Different Speeds in Persons With Haemophilia

**DOI:** 10.1111/hae.70305

**Published:** 2026-05-07

**Authors:** Marius Brühl, Pia Möllers, Jamil Hmida, Fabian Tomschi, Georg Goldmann, Frank A. Schildberg, Johannes Oldenburg, Andreas C. Strauss, Thomas Hilberg

**Affiliations:** ^1^ Department of Sports Medicine University of Wuppertal Wuppertal Germany; ^2^ Department of Orthopaedics and Trauma Surgery University of Bonn Bonn Germany; ^3^ Institute For Experimental Haematology and Transfusion Medicine University of Bonn Bonn Germany

**Keywords:** arthropathy, gait, joint, haemophilia, motion capture

## Abstract

**Introduction:**

Persons with haemophilia (PwH) have a risk of bleeding in joints, especially in elbow, knee and ankle. In the long term, this leads to haemophilic arthropathy (HA), which results in joint deformities.

**Aim:**

This study aims to examine how walking speed and HA affect (1) foot pressure distribution, (2) average vertical peak pressure, and (3) knee and ankle joint angles during walking.

**Methods:**

Using a motion analysis system (DIERS 4DmotionLab), 30 PwH and 31 healthy controls (CG) were examined at speeds of 3, 4, and 5 km/h. Groups were additionally divided according to the severity of HA, in the subgroups PwH(major) and PwH(minor).

**Results:**

Regarding different gait speeds, no differences were found between PwH and CG in peak pressure distribution. However, pedobarography showed a significant reduction in average vertical peak pressure in the second half of the stance phase in PwH(major) compared to CG (*p* < 0.007). Joint angle measurements showed reduced plantar flexion (*p* = 0.041), dorsiflexion (*p* = 0.011), and descriptive, non‐significant knee flexion in PwH(major) at different speeds. Knee extension did not differ between groups.

**Conclusion:**

The study confirms that PwH, especially those with advanced HA, show impaired gait pattern. While different walking speeds had no impact on peak pressure values compared to CG, force transmission was reduced in PwH in the second half of the stance phase. This, in conjunction with reduced mobility in the ankle joints, indicates impaired power transmission in the propulsion phase which is well compensated.

## 1 Introduction

In persons with haemophilia (PwH), recurrent bleeding into a joint (haemarthrosis) leads to inflammation and damage to the joint cartilage and synovial membrane (synovitis), which over time leads to joint deformities, pain and restricted movement [1]. This condition is defined as haemophilic arthropathy (HA), in which mostly large joints such as ankle (41%), knee (27%), and elbow (11%) are affected [2].

These HA have a variety of effects and influence gait, among others. For example, it was shown that PwH expend more metabolic energy to walk depending on their joint health [3]. Biomechanically, a slower gait speed and increased cadence were also reported compared to CG [4]. In addition, the pressure distribution during walking at 3 km/h was altered. The peak pressure in metatarsals II‐IV and the heel was significantly reduced in some cases [5].

Due to a high heterogeneity of the HA within PwH, intragroup comparability is limited. Furthermore, the gait speed of 3 km/h chosen in the aforementioned study represents a rather slow gait and could underchallenge PwH with mild HA. Studies have shown in clinical tests such as the 50 metre walk test and the 4 metre walk test that PwH walked significantly slower than CG [6, 7]. Tomschi et al. reported a simulated usual pace over 4 metres for PwH of 1.0 metre per second (3.6 km/h) and 1.2 metre per second (4.3 km/h) for CG [6]. Progressively increasing the walking speed from 3 to 4 up to 5 km/h presents a valuable opportunity to impose greater physical demands on participants and thereby reinforcing modifications in gait patterns. HA is known to be a common cause of gait changes in PwH due to reduced joint mobility of the knee and ankle joints in addition to pain [5]. However, it should be noted that these observations relate to complete joint angle measurements of the maximum possible flexion and extension, which are measured as part of the haemophilia‐specific clinical examination (Haemophilia Joint Health Score, HJHS) and not specifically in the gait. There is a lack of knowledge in how far the reduced joint mobility influences the gait pattern and in how far PwH show altered joint angles during walking.

Based on the above mentioned considerations, this study aims to (1) investigate the influence of different walking speeds on pressure distribution of individual foot areas in PwH depending on HA; (2) examine the average vertical peak pressure values depending on HA and speed; (3) compare the knee and ankle joint angles measured by motion capture during walking depending on the HA and speed.

## 2 Material and Methods

### 2.1 Study Design

This study was designed as a cross‐sectional case‐control study and the reporting of this study is performed according to the STROBE checklist for case‐controlled studies [8]. The study was conducted in accordance with the declaration of Helsinki and the study protocol was approved by the ethics committee of the Medical Faculty Bonn (329/19).

### 2.2 Setting

The study took place from April 2021 to December 2022 at the University of Bonn in the Department of Orthopedics and Surgery. PwH were recruited in cooperation with the Institute for Experimental Haematology and Transfusion Medicine. CG was recruited via announcements.

After providing written informed consent, participants completed a medical history questionnaire to check their eligibility for inclusion and undressed down to their underwear for the clinical joint examination.

Afterwards, the gait analysis was prepared according to the guidelines of the manufacturer DIERS (Schlangenbad, Germany), which required the participant to undress down to his underpants and a total of 10 anatomical landmarks (Trochanter major, lateral joint space knee centre, malleolus lateralis, calcaneus leading edge, and metatarsal head V) was marked with 1 cm^2^ reflective markers. The participant then stood barefoot on the treadmill, where the standardized study protocol began. First, the treadmill started at 3 km/h and the participant was given a mandatory familiarization period of 2 min [9]. After the 2‐min familiarization period, the 6‐s measurement was started without interruption. The treadmill then stopped automatically, and the participant had to leave the treadmill for 2 min to save the measured data and calibrate the measuring platform. During this break, the participants sat down on a chair so as not to experience any additional physical strain. The same procedure including familiarization was repeated at 4 and 5 km/h.

### 2.3 Participants

A total of 61 male subjects were recruited in this study. 30 were PwH and 31 CG. The sample size was calculated a priori using G*Power version 3.1 (Düsseldorf, Germany) based on previous studies with a medium effect of *f* = 0.25, a power analysis showed that 54 participants were needed for 0.95 power at *α* = 0.05. With an expected 10% dropout rate of, a target of 60 participants was set.

Included were adults (≥18 years) with severe haemophilia A or B and BMI and age‐matched healthy men as controls. Exclusion criteria were other bleeding disorders, bleeding events in the last two weeks, inhibitors, joint surgery, spinal surgery, spinal disorders such as Scheuermann's disease, acute musculoskeletal injuries, and inability to walk without walking frames, and for the CG additionally osteoarthritis or rheumatoid arthritis.

### 2.4 Clinical Joint Examination

Orthopaedic joint status was determined using the Haemophilia Joint Health Score v2.1. Hereby, ankles, knees, and elbows are evaluated regarding axial deformity, muscle atrophy, swelling, range of motion (extension and flexion) using goniometer, crepitus of motion, joint pain in the past two weeks, and strength. The result of this examination is a score ranging from 0 (no HA) to 124 (most pronounced HA) [10].

### 2.5 Gait Analysis

Participants underwent gait analysis using the DIERS 4DmotionLab system and Dicam v3.18 software (DIERS International GmbH, Germany) [11]. The pressure distribution was measured in N/cm^2^ using a capacitive pressure plate integrated into the Zebris FDM‐T treadmill. (Zebris medical GmbH, Germany). The plate (1084 × 474 mm) contains 7186 sensors (1.4 sensors/cm^2^), records at 120 Hz, measures 1–120 N/cm^2^ ± 5%, has a 1 N/cm^2^ threshold, and <3% hysteresis. It is widely used in gait studies to assess foot pressure and has repeatedly shown high reliability [12].

### 2.6 Statistics

Statistical analyses were performed using the IBM SPSS 29 software (Armonk, NY, USA) for macOS. The maximum pressure values and joint angles of the right and left limbs were included separately in the analysis. To normalize the distribution, a log10 transformation of the data set was performed and a two‐way variance analysis with the factors ‚speed‘ and ‚group’ with Bonferroni correction was used. A significance level of *p* ≤ 0.05 with a confidence interval of 95% was set.

**FIGURE 1:**
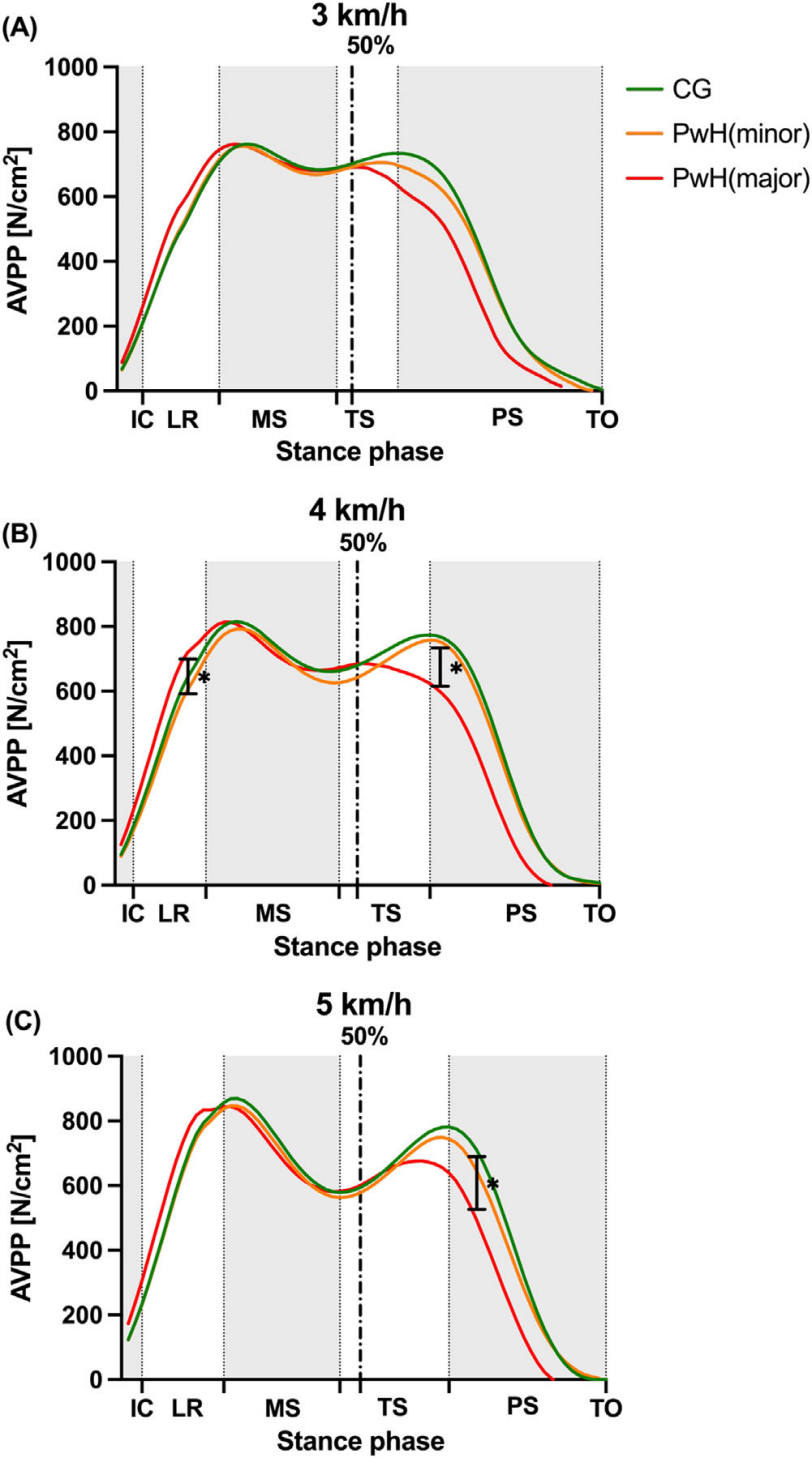
Visualisation of the average vertical peak pressure values in N/cm^2^ during the stance phase of all gait cycles at (A) 3, (B) 4, and (C) 5 km/h. AVPP, average vertical peak pressure values; IC, initial contact; LR, loading response; MS, midstance; TS, terminal stance; PS, pre‐swing; TO, toe off; *, *p* ≤ 0.05 PwH(major) vs. CG.

Graphs were plotted using GraphPad Prism 10 (Boston, Massachusetts, USA). With this program, the smoothing function was used in Figure 1 (creation of 4 data neighbours on each side) to create visually recognisable lines that depict the average vertical peak pressure values (AVPP) in N/cm^2^ during the stance phase of all gait cycles. This procedure had no influence on the statistical analysis. To compare statistically the different AVPP of the groups in stance phase shown in Figure 1, the stance phase was divided at 50% and the AVPP were compared using ANOVA. The first half represents the loading response and early midstance phase. The second half represents the late midstance, terminal stance, and pre‐swing phase. Data are presented as mean ± standard deviation (minimum—maximum).

## 3 Results

### 3.1 Participants

30 PwH and 31 CG were included in this study. All participants were able to walk at all speeds resulting in no dropouts or missing data.

Anthropometric data are displayed in Table 1. The groups did not differ in age, height, weight or BMI (*p* > 0.05).

**TABLE 1:** Comparison of anthropometric data between (A) PwH and CG; (B) PwH(major), PwH(minor) and CG.

(A)			
Parameter	PwH (*n* = 30)	CG (*n* = 31)	*p* value
Age (years)	41.3 ± 11.8 (22–66)	41.4 ± 12.7 (25–61)	0.891
Weight (kg)	82.5 ± 12.0 (62.0–113.0)	84.7 ± 10.7 (69.0–110.0)	0.431
Height (m)	1.79 ± 0.53 (1.67–1.87)	1.82 ± 0.06 (1.67–1.98)	0.076
Body mass Index (kg/m^2^)	25.7 ± 3.4 (20.2–35.7)	25.6 ± 3.0 (20.8–35.1)	0.784
HJHS total (score points)	19.8 ± 16.1 (0.0–62.0)	4.5 ± 3.5 (0.0–11.0)	<0.001
HJHS sum of the lower extremity	13.2 ± 11.3 (0.0–44.0)	2.8 ± 2.1 (0.0–7.0)	<0.001
NRS joint past 2 weeks	3.0 ± 2.4 (0.0–9.0)	0.7 ± 1.3 (0.0–5.0)	<0.001
Type of haemophilia (*n*)	A: 25 B: 5	—	—
HIV (*n*)	Yes: 5 No: 25	Yes: 0 No: 31	—
Hepatitis (*n*)	Yes 8 No: 22	Yes: 0 No: 31	—

(B)				
Parameter	PwH(major) (*n* = 15)	PwH(minor) (*n* = 15)	CG (*n* = 31)	*p* value
Age (years)	47.6 ± 12.0 (28–66)^#^	34.9 ± 7.7 (22–52)	41.4 ± 12.7 (25–61)	0.028
Weight (kg)	82.5 ± 14.6 (62.0–113.0)	82.5 ± 9.4 (68.0–103.0)	84.7 ± 10.7 (69.0–110.0)	0.733
Height (*m*)	1.78 ± 0.06 (1.67–1.87)	1.80 ± 0.05 (1.71–1.87)	1.82 ± 0.06 (1.67–1.98)	0.169
Body mass Index (kg/m^2^)	25.9 ± 3.9 (20.2–35.7)	25.5 ± 2.8 (21.0–31.8)	25.6 ± 3.0 (20.8–35.1)	0.963
HJHS total (score points)	32.2 ± 13.8 (13.0–62.0)^*^ ^#^	7.3 ± 3.6 (0.0–12.0)	4.5 ± 3.5 (0.0–11.0)	<0.001
HJHS sum of the lower extremity	22.3 ± 8.9 (10.0–44.0)^*^ ^#^	4.1 ± 3.1 (0.0–8.0)	2.8 ± 2.1 (0.0–7.0)	<0.001
NRS joint past 2 weeks	3.7 ± 2.5 (0.0–9.0)^*^	2.4 ± 2.2 (0.0–6.0)^*^	0.7 ± 1.3 (0.0–5.0)	<0.001
Type of haemophilia (*n*)	A: 12 B: 3	A: 12 B: 2	—	—
HIV (*n*)	Yes: 5 No: 10	Yes: 0 No: 15	Yes: 0 No: 31	—
Hepatitis (*n*)	Yes: 7 No: 8	Yes: 1 No: 14	Yes: 0 No: 31	—

*Note*: Data presented as mean ± standard deviation (min–max).

Abbreviations: PwH, persons with haemophilia; CG, control group; HJHS, haemophilia joint health score; NRS, numeric rating scale.

* *p* ≥ 0.05 vs. CG.

^#^ *p* ≤ 0.05 vs. PwH(minor).

For further analysis, subgroups were defined based on the HJHS of the lower extremities to assess the high heterogeneity of HA within the PwH, as done before [5]. The total score for both ankle and knee joints was calculated for each PwH, and then the 50% of subjects with the lower total score were sorted into the PwH(minor) group and the 50% of subjects with the higher total score were sorted into the PwH(major) group. This resulted in the subgroups PwH(major), with more severe HA (*n* = 15) (HJHS of the lower extremities: 22.3 ± 8.9 (10–44)) and PwH(minor), with milder HA (*n* = 15) (HJHS of the lower extremities: 4.1 ± 3.1 (0–8)).

### 3.2 Pedobarography

The maximum pressure values in N/cm^2^ in the individual areas of the foot at 3, 4 and, 5 km/h are presented in Table 2A. No significant main or interaction effects were observed between PwH and CG nor between the groups depending on the walked speed 3, 4, and 5 km/h. The same results were observed for the subgroups PwH(major) and PwH(minor) (Table 2B).

**TABLE 2:** Comparison of maximum pressures in the individual foot areas at different speeds between (A) PwH and CG; (B) PwH(major), PwH(minor) and CG.

(A)						
Parameter	Gait speed	PwH (*n* = 30)	CG (*n* = 31)	Main effect (group)	Main effect (speed)	Interaction effect (groupxspeed)
**Heel medial** **max. (N/cm^2^)**	**3 km/h**	23.4 ± 5.3 (12.5–37.5)	24.0 ± 4.2 (14.5–33.0)	0.299	<0.001	0.989
	**4 km/h**	27.8 ± 5.5 (19.0–42.0)	28.4 ± 4.0 (20.0–38.0)			
	**5 km/h**	32.2 ± 8.0 (4.5–50.5)	32.1 ± 5.6 (18.5–48.0)			
**Heel lateral** **max. (N/cm^2^)**	**3 km/h**	22.8 ± 5.3 (12.5–37.0)	23.1 ± 4.0 (15.5–31.5)	0.701	<0.001	0.709
	**4 km/h**	27.4 ± 5.8 (18.0–41.0)	26.7 ± 4.8 (12.0–41.0)			
	**5 km/h**	32.6 ± 7.9 (14.0–54.5)	31.7 ± 6.1 (20.0–50.0)			
**Midfoot** **max. (N/cm^2^)**	**3 km/h**	8.9 ± 4.0 (4.0–24.5)	9.6 ± 5.5 (2.0–32.5)	0.452	0.010	0.954
	**4 km/h**	10.1 ± 6.4 (3.5–49.0)	10.4 ± 4.7 (3.0–26.5)			
	**5 km/h**	9.9 ± 4.7 (3.0–28.0)	10.0 ± 3.7 (4.5–23.5)			
**Forefoot medial** **max. (N/cm^2^)**	**3 km/h**	29.2 ± 13.0 (6.5–67.0)	28.2 ± 12.5 (2.5–53.0)	0.257	<0.007	0.895
	**4 km/h**	33.9 ± 17.0 (1.5–70.0)	30.2 ± 16.8 (1.0–81.0)			
	**5 km/h**	38.8 ± 16.4 (2.0–83.5)	34.6 ± 15.0 (1.0–75.5)			
**Forefoot lateral** **max. (N/cm^2^)**	**3 km/h**	20.2 ± 15.3 (0.0–59.5)	20.4 ± 14.0 (0.0–48.0)	0.561	0.019	0.335
	**4 km/h**	23.6 ± 17.1 (1.0–69.5)	23.1 ± 16.5 (1.5–77.5)			
	**5 km/h**	24.6 ± 18.7 (1.5–62.5)	24.8 ± 16.0 (3.5–56.5)			
**Toes** **max. (N/cm^2^)**	**3 km/h**	6.8 ± 3.9 (1.0–23.0)	6.4 ± 4.3 (3.0–35.0)	0.232	<0.001	0.145
	**4 km/h**	7.6 ± 3.2 (2.5–17.0)	8.5 ± 4.2 (3.0–25.0)			
	**5 km/h**	8.1 ± 2.8 (2.5–14.5)	9.8 ± 4.8 (3.5–27.5)			
**Hallux** **max. (N/cm^2^)**	**3 km/h**	25.9 ± 13.1 (0.0–54.5)	24.2 ± 12.3 (4.5–53.0)	0.493	<0.001	0.424
	**4 km/h**	29.9 ± 17.2 (5.0–70.0)	29.4 ± 17.4 (4.0–81.0)			
	**5 km/h**	35.4 ± 17.4 (8.5–83.5)	32.1 ± 15.2 (0.0–75.5)			

(B)							
Parameter	Gait speed	PwH(major) (*n* = 15)	PwH(minor) (*n* = 15)	CG (*n* = 31)	Main effect (group)	Main effect (speed)	Interaction effect (groupxspeed)
**Heel medial** **max. (N/cm^2^)**	**3 km/h**	22.7 ± 6.0 (12.5–37.5)	24.0 ± 4.6 (15.5–31.0)	24.0 ± 4.2 (14.5–33.0)	0.548	<0.001	0.187
	**4 km/h**	29.2 ± 5.9 (19.5–42.0)	26.3 ± 4.7 (19.0–38.5)	28.4 ± 4.0 (20.0–38.0)			
	**5 km/h**	33.3 ± 9.4 (4.5–50.5)	31.1 ± 6.0 (14.0–42.0)	32.1 ± 5.6 (18.5–48.0)			
**Heel lateral max. (N/cm^2^)**	**3 km/h**	22.1 ± 5.9 (12.5–37.0)	23.4 ± 4.5 (15.5–31.0)	23.1 ± 4.0 (15.5–31.5)	0.817	<0.001	0.069
	**4 km/h**	28.7 ± 6.3 (18.5–41.0)	26.0 ± 5.0 (18.0–38.5)	26.7 ± 4.8 (12.0–41.0)			
	**5 km/h**	33.7 ± 8.9 (15.0–54.5)	31.4 ± 6.5 (14.0‐42.5)	31.7 ± 6.1 (20.0–50.0)			
**Midfoot max. (N/cm^2^)**	**3 km/h**	9.2 ± 3.7 (5.0–24.5)	8.5 ± 4.3 (4.0‐23.0)	9.6 ± 5.5 (2.0–32.5)	0.248	0.018	0.947
	**4 km/h**	11.0 ± 8.3 (3.5–49.0)	9.1 ± 3.5 (3.5‐18.5)	10.4 ± 4.7 (3.0–26.5)			
	**5 km/h**	10.5 ± 5.5 (3.0–28.0)	9.3 ± 3.7 (3.0–19.5)	10.0 ± 3.7 (4.5–23.5)			
**Forefoot medial max. (N/cm^2^)**	**3 km/h**	30.5 ± 12.7 (7.5–54.5)	27.9 ± 13.4 (6.5–67.0)	28.2 ± 12.5 (2.5–53.0)	0.524	0.008	0.848
	**4 km/h**	31.5 ± 16.5 (1.5–65.0)	36.5 ± 17.3 (1.5–70.0)	30.2 ± 16.8 (1.0–81.0)			
	**5 km/h**	38.8 ± 16.3 (2.0–78.0)	38.1 ± 16.7 (5.5–83.5)	34.6 ± 15.0 (1.0–75.5)			
**Forefoot lateral max. (N/cm^2^)**	**3 km/h**	17.2 ± 12.6 (0.0–41.0)	23.3 ± 17.3 (0.0–59.5)	20.4 ± 14.0 (0.0–48.0)	0.459	0.057	0.277
	**4 km/h**	22.6 ± 19.3 (1.0–69.5)	24.7 ± 14.7 (4.0–57.0)	23.1 ± 16.5 (1.5–77.5)			
	**5 km/h**	24.4 ± 18.4 (1.5–62.5)	24.8 ± 19.8 (2.0–56.5)	24.8 ± 16.0 (3.5–56.5)			
**Toes max. (N/cm^2^)**	**3 km/h**	7.9 ± 4.5 (3.0–23.0)	5.6 ± 2.9 (1.0–14.0)	6.4 ± 4.3 (3.0–35.0)	0.332	<0.001	0.222
	**4 km/h**	7.6 ± 3.6 (2.5–17.0)	7.6 ± 2.6 (3.5–14.0)	8.5 ± 4.2 (3.0–25.0)			
	**5 km/h**	8.0 ± 3.0 (2.5–14.5)	8.2 ± 2.7 (3.5–14.5)	9.8 ± 4.8 (3.5–27.5)			
**Hallux max. (N/cm^2^)**	**3 km/h**	28.7 ± 12.1 (7.5–54.5)	23.1 ± 13.5 (0.0–47.5)	24.2 ± 12.3 (4.5–53.0)	0.785	<0.001	0.299
	**4 km/h**	29.0 ± 18.1 (5.0–69.5)	30.8 ± 16.6 (7.0–70.0)	29.4 ± 17.4 (4.0–81.0)			
	**5 km/h**	34.0 ± 17.7 (8.5–78.0)	37.0 ± 17.1 (9.5–83.5)	32.1 ± 15.2 (0.0–75.5)			

*Note*: Data presented as mean ± standard deviation (min–max).

Abbreviations: PwH, persons with haemophilia; CG, control group.

To evaluate the pressure distribution more holistically, the stance phase was divided at 50% and the AVPP in N/cm^2^ were compared between subgroups. No significant difference were found at 3 km/h (Figure 1A). At 4 km/h, PwH(major) showed a significantly higher AVPP in the first half of the stance phase compared to CG (*p* = 0.039) and PwH(minor) (*p* < 0.001), and a reduced value in the second half compared to CG (*p* = 0.003) (Figure 1B). At 5 km/h, PwH(major) again showed a significantly reduced AVPP in the second half compared to CG (*p* = 0.007) (Figure 1C).

### 3.3 Joint Angles

For the analysis of the joint angles during walking, the total PwH group and the subgroups PwH(major) and PwH(minor) were compared to CG.

The statistical analysis of the maximum angles of the knee joint showed no difference in knee extension and flexion compared to CG. Neither in the total PwH group (Table 3A), nor in the subgroups PwH(major) and PwH(minor) (Table 3B).

**TABLE 3:** Comparison of joint angles while walking at different speeds between (A) PwH and CG; (B) PwH(major), PwH(minor) and CG.

(A)						
Parameter	Gait speed	PwH (*n* = 30)	CG (*n* = 31)	Main effect (group)	Main effect (speed)	Interaction effect (groupxspeed)
**max. Knee** **extension (°)**	**3 km/h**	1.8 ± 6.1 (−11.0–20.0)	1.8 ± 5.9 (−17.0–14.0)	0.719	0.511	0.137
	**4 km/h**	1.1 ± 6.0 (−13.0–17.0)	2.1 ± 4.8 (−8.0–13.0)			
	**5 km/h**	1.7 ± 5.8 (−14–19)	1.7 ± 4.5 (−7.0–11.0)			
**max. Knee** **flexion (°)**	**3 km/h**	60.3 ± 5.9 (42.0–74.0)	61.1 ± 7.3 (15.0–69.0)	0.302	<0.001	0.386
	**4 km/h**	62.3 ± 6.2 (32.0–73.0)	64.1 ± 4.6 (54.0–72.0)			
	**5 km/h**	63.9 ± 6.7 (32.0–81.0)	64.9 ± 4.5 (54.0–74.0)			
**max. Ankle** **Dorsiflexion (°)**	**3 km/h**	1.6 ± 4.1 (−8.0–14.0)	3.6 ± 5.1 (−10.0–15.0)	0.006	0.688	0.110
	**4 km/h**	1.0 ± 4.7 (−11.0–11.0)^*^	3.4 ± 3.5 (−4.0–11.0)			
	**5 km/h**	1.2 ± 4.4 (−9.0–13.0)^*^	3.2 ± 3.9 (−7.0–12.0)			
**max. Ankle** **Plantar flexion (°)**	**3 km/h**	19.6 ± 5.8 (9.0–34.0)	20.6 ± 7.7 (6.0–44.0)	0.111	<0.001	0.047
	**4 km/h**	23.0 ± 6.8 (7.0–38.0)^*^	26.0 ± 7.5 (13.0–48.0)			
	**5 km/h**	26.8 ± 7.1 (11.0–43.0)	29.0 ± 7.3 (14.0–49.0)			

(B)							
Parameter	Gait speed	PwH(major) (*n* = 15)	PwH(minor) (*n* = 15)	CG (*n* = 31)	Main effect (group)	Main effect (speed)	Interaction effect (groupxspeed)
**max. Knee** **extension (°)**	**3 km/h**	1.4 ± 5.5 (−11.0–17.0)	2.2 ± 6.7 (−9.0–20.0)	1.8 ± 5.9 (−17.0–14.0)	0.774	0.937	0.235
	**4 km/h**	1.3 ± 5.7 (−13.0–17.0)	1.0 ± 6.4 (−9.0–17.0)	2.1 ± 4.8 (−8.0–13.0)			
	**5 km/h**	1.5 ± 5.0 (−14.0–16.0)	2.0 ± 6.6 (−10.0–19.0)	1.7 ± 4.5 (−7.0–11.0)			
**max. Knee flexion (°)**	**3 km/h**	59.1 ± 5.9 (42.0–67.0)	61.5 ± 5.8 (53.0–74.0)	61.1 ± 7.3 (15.0–69.0)	0.096	<0.001	0.415
	**4 km/h**	61.5 ± 7.7 (32.0–73.0)	63.1 ± 4.2 (56.0–71.0)	64.1 ± 4.6 (54.0–72.0)			
	**5 km/h**	61.7 ± 7.2 (32.0–71.0)	66.0 ± 5.5 (59.0–81.0)	64.9 ± 4.5 (54.0–74.0)			
**max. Ankle Dorsiflexion (°)**	**3 km/h**	0.5 ± 3.7 (−8.0–8.0)	2.6 ± 4.3 (−5.0–14.0)	3.6 ± 5.1 (−10.0–15.0)	0.011	0.345	0.098
	**4 km/h**	0.5 ± 4.2 (−8–11.0)^*^	1.6 ± 5.3 (−11.0–11.0)^*^	3.4 ± 3.5 (−4.0–11.0)			
	**5 km/h**	0.2 ± 3.4 (−7–10.0)^*^	2.2 ± 5.0 (−9.0–13.0)	3.2 ± 3.9 (−7.0–12.0)			
**max. Ankle Plantar flexion (°)**	**3 km/h**	18.7 ± 5.8 (9.0–32.0)	20.5 ± 5.7 (12.0–34.0)	20.6 ± 7.7 (6.0–44.0)	0.041	<0.001	0.076
	**4 km/h**	21.2 ± 7.3 (7.0–36.0)^*^, ^#^	24.8 ± 6.0 (14.0–38.0)	26.0 ± 7.5 (13.0–48.0)			
	**5 km/h**	25.3 ± 7.8 (14.0–43.0)^*^	28.3 ± 6.1 (11.0–39.0)	29.0 ± 7.3 (14.0–49.0)			

*Note*: Data presented as mean ± standard deviation (min–max).

Abbreviations: PwH, persons with haemophilia; CG, control group.

* *p* ≤ 0.05 vs. CG.

^#^ *p* ≤ 0.05 vs. PwH(minor).

At the ankle joint, main effects between PwH and CG for the factor ‘group’ were observed for dorsiflexion (*p* = 0.006) and plantar flexion for the ‘groupxspeed’ interaction (*p* = 0.047). Post hoc analyses showed a significantly lower dorsiflexion angle in PwH at 4 and 5 km/h and a lower plantar flexion at 4 km/h compared to CG. The analysis of the subgroups strengthens these results of reduced angles in PwH(major) in dorsiflexion (*p* = 0.011) and plantar flexion (*p* = 0.041). Post hoc shows that both dorsiflexion and plantar flexion are reduced in PwH(major) at 4 and 5 km/h compared to CG. In addition, PwH(minor) also showed less dorsiflexion than CG at 4 km/h and PwH(major) showed less plantar flexion than PwH(minor) at 4 km/h.

## 4 Discussion

Gait analysis is essential in locomotion research, particularly in PwH. Arthropathic changes in the knee and ankle joints represent a relevant biomechanical research approach. These changes can lead to restricted movement, pain, and a disturbed gait pattern, which might impair the quality of life of those affected [5, 13].

The central study aims are addressed in the following discussion:

(1) to investigate the influence of different walking speeds on pressure distribution of individual foot areas in PwH depending on HA; (2) examine the average vertical peak pressure values depending on HA; (3) compare the knee and ankle joint angles measured by motion capture during walking depending on the HA.

The analyses showed no differences in pressure distribution in the individual areas of the foot, but a reduced AVPP in the second half of the stance phase in PwH(major). In addition, PwH(major) had reduced joint angles in the ankle joints but not in the knee joints. These results will be discussed and interpreted below. It was shown that there were no group differences in the individual foot areas of maximum pressure at any of the measured speeds (3, 4, and 5 km/h) (Table 2A). Although it was shown that the measured pressure increased significantly with increasing speed in all foot areas (*p* = 0.019 ‐ <0.001), except the forefoot lateral in the subgroups (*p* = 0.057), this was equally the same in PwH(major), PwH(minor), and CG (Table 2B). Thus, the previously reported results of an increased peak pressure value in metatarsals II‐IV and reduced peak pressure value in the heel could not be confirmed [5].

However, the analysis of the AVPP during the stance phase showed increased AVPP in the first half and reduced AVPP in the second half of the stance phase at 4 km/h in PwH(major) to CG (Figure 1B). At 5 km/h, this change was observed only in the second half of the stance phase (Figure 1C).

It is known that joint mobility is reduced in PwH due to HA [14]. These findings relate to the maximum possible extension and flexion of the respective joint, which is part of the HJHS, but only allow a limited statement to be made about the required joint angles during walking.

To close this gap, motion capture was used, which showed that there was no statistically significant group difference in knee flexion or extension at any speed between the groups. However, PwH(major) indicated a non‐significant but descriptively lower knee flexion than PwH(minor) and CG, which increased at higher speeds (Table 3B). It can be assumed that PwH(minor) and CG increase knee flexion in the load‐free initial swing phase with increasing speed, which is not observed in PwH(major). The maximum knee extension assumed in the loading response and midstance phase [15] does not differ in any of the groups or across speeds. This leads to the assumption that the knee joint in PwH(major) only shows changes in joint angles at increased speeds in flexion but not in extension.

The ankle joint, which is considered the most affected joint in HA [2], showed reduced joint angles in dorsiflexion but also in plantar flexion in PwH compared to CG. Maximum dorsiflexion is required in the transition from the late midstance phase to the terminal stance phase and serves to transfer force for pushing off and gaining forward propulsion [16]. Here, a lower maximum joint angle at PwH to CG can be seen. Maximum plantar flexion is required in the forward propulsion, which follows immediately after the terminal stance phase [15]. A reduced joint angle between PwH and CG was also observed. Consideration of the PwH(major) and PwH(minor) subgroups (Table 3B) indicates that even PwH(minor) already show an altered ankle joint angle during walking, which appears to be even more pronounced in PwH(major).

This is a novel finding with this measurement method and indicates that loss of mobility in the ankle joint caused by HA lead to a disturbed gait pattern. These results of the measured joint angles can be cautiously interpreted in conjunction with the reported altered AVPP in the stance phase (Figure 1). This shows that the second half of the stance phase, which represents the end of the midstance, terminal stance, and pre‐swing phase, PwH(major) exerts a reduced AVPP compared to CG. The reason for this seems to be the limited dorsiflexion, plantar flexion, and strength in PwH, which are required in the late midstance, terminal stance, and pre‐swing phase. This leads to a reduced ability to generate forward propulsion.

The results of this study are clinically relevant, as altered gait in terms of peak pressure values and joint mobility was observed in PwH, most likely due to advanced HA. Despite multiple joint HA and pain in PwH, the differences in gait analysis compared to CG were not as severe as expected. Detailed analysis of the subgroups in PwH(major) and PwH(minor) provides deeper insights and shows that the severity of HA is a significant influencing factor. Furthermore, PwH appear to have developed good compensatory strategies to maintain gait. This compensation could be the reason why PwH expend more metabolic energy during walking, as their gait is more inefficient [3]; however, this was not measured in this study and can therefore only be assumed.

Nonetheless, maintaining ankle joint mobility for as long as possible should be the primary therapeutic goal. Consisting of low‐impact sports, physiotherapy, and regular routine checks of the joints, is key to long‐term joint health. Early detection and treatment of synovitis, which is considered a precursor to HA [17], should be routinely performed at an early stage. In the case of already present severe HA, these patients will certainly benefit from adapted sports and physiotherapy but will experience little improvement in joint mobility. For these severely affected patients, pain relief is the primary therapeutic goal. This is sometimes necessary through surgical intervention, such as ankle arthrodesis, after which mobility is completely lost [18]. Less often, total ankle replacement is also performed as an alternative. It offers the benefit that, in addition to pain reduction, increased mobility of the joint can be maintained or regained [18], which may be relevant for everyday gait. However, there is a lack of long‐term studies on the long‐term survival rate of implants following total ankle replacement, which makes it difficult to provide a recommendation at this time [18].

## 5 Limitations

The human gait is affected by many intra‐individual variables, which makes comparability difficult. In the chosen study design, standardized speeds were selected to ensure better comparability and uniform load. However, the used approach could have led to individual participants not walking at their usual pace. Furthermore, walking on the treadmill is unusual for some participants, which could also have led to distortions. Yet, all participants managed to walk at 5 km/h. Due to the different constitutions of the participants, they were challenged to different degrees at the defined speeds, so that no uniform state of fatigue was produced. Furthermore, the measurement of maximum joint angles provides only limited insight into the differentiation of the individual gait phases, as this classification was not part of the analysis. Future work could increase pre‐fatigue by, letting the participants walk for a longer period at the selected speed and measure them immediately afterwards.

## 6 Conclusion

The results confirm that PwH, especially those with severe HA, show significant gait alterations. PwH showed no differences in peak pressure values of the individual foot areas compared to CG at different speeds. However, in the AVPP, a significantly higher force transmission was observed in PwH(major) in the first half of the stance phase and a significantly lower force transmission in the second half of the stance phase. Additionally, PwH showed lower mobility in the ankle joint in dorsiflexion and plantar flexion, when walking at all speeds and tends to have lower knee flexion. This in combination with reduced AVPP in the second half of the stance phase, which represents the late midstance, terminal stance, and the pre‐swing phase, demonstrates the impaired gait pattern in PwH.

## Author Contributions

Jamil Hmida and Thomas Hilberg had initial idea of the study. Jamil Hmida, Marius Brühl, Thomas Hilberg, and Andreas C. Strauss designed the study, Marius Brühl, Pia Möllers, and Jamil Hmida performed the data collection. Marius Brühl and Fabian Tomschi analysed the results. Marius Brühl wrote the first draft of the paper. Thomas Hilberg, Andreas C. Strauss, Fabian Tomschi, Frank A. Schildberg, and Pia Möllers edited the manuscript. J. Oldenburg, Georg Goldmann and Andreas C. Strauss conducted the participant acquisition. Andreas C. Strauss and Thomas Hilberg supervised the project.

## Funding

The authors have nothing to report.

## Ethics Statement

The study protocol was approved by the local Ethics Committee (Friedrich–Wilhelms–Universität Bonn, 329/19) and was conducted in accordance with the Declaration of Helsinki. All participants provided written informed consent.

## Conflicts of Interest

Marius Brühl has received travel support from Swedish Orphan Biovitrum (Sobi) and Takeda, and speaker's fees from Takeda. Pia Möllers, Jamil Hmida, and Frank A. Schildberg declare no conflicts of interest. Fabian Tomschi has received speaker's fees and travel support from Takeda and an educational grant from Sobi. Georg Goldmann has received honoraria (lectures, presentations, speaker's bureau, educational events) from Sobi, Bayer, Takeda, Octapharma, Novo Nordisk, Biotest, and Roche, and travel support from Sobi, Biotest, and Novo Nordisk. J. Oldenburg has received research funding from Bayer, Biotest, CSL Behring, Octapharma, Pfizer, Sobi, and Takeda, and consultancy, speaker's bureau, advisory board honoraria, and travel support from Bayer, Biogen Idec, BioMarin, Biotest, Chugai, CSL Behring, Freeline, Grifols, LFB, Novo Nordisk, Octapharma, Pfizer, Roche, Sanofi, Spark Therapeutics, Sobi, and Takeda. Andreas C. Strauss has received consulting fees from Sobi, Bayer, Takeda, and CSL Behring, and research funding from Sobi, Bayer, and Bauerfeind AG. Thomas Hilberg has received research funding from Biotest, Chugai, Novo Nordisk, Intersero, Roche, Sobi, and Takeda, and travel support or honoraria (speaker/advisory board) from Bayer, Chugai, Novo Nordisk, Pfizer, Roche, Sanofi, Sobi, and Takeda.

## Data Availability

The data that support the findings of this study are available from the corresponding author upon reasonable request.
